# Extracting reproducible subject-specific MEG evoked responses with independent component analysis

**DOI:** 10.1162/imag_a_00182

**Published:** 2024-06-05

**Authors:** Silvia Federica Cotroneo, Heidi Ala-Salomäki, Lauri Parkkonen, Mia Liljeström, Riitta Salmelin

**Affiliations:** Department of Neuroscience and Biomedical Engineering, Aalto University, Espoo, Finland; Aalto NeuroImaging, Aalto University School of Science, Aalto, Finland; BioMag Laboratory, HUS Medical Imaging Center, Helsinki University Hospital, Helsinki, Finland

**Keywords:** independent component analysis, magnetoencephalography, individual-level, reproducibility

## Abstract

Reliable individual-level measures of neural activity are essential for capturing interindividual variability in brain activity recorded by magnetoencephalography (MEG). While conventional group-level analyses highlight shared features in the data, individual-level specificity is often lost. Current methods for assessing reproducibility of brain responses focus on group-level statistics and neglect subject-specific temporal and spatial characteristics. This study proposes a combined ICA algorithm (comICA), aimed at extracting within-individual consistent MEG evoked responses. The proposed hypotheses behind comICA are based on the temporal profiles of the evoked responses, the corresponding spatial information, as well as independence and linearity. ComICA is presented and tested against simulated data and test–retest recordings of a high-level cognitive task (picture naming). The results show high reliability in extracting the shared activations in the simulations (success rate >93%) and the ability to successfully reproduce group-level results on reproducibility for the test–retest MEG recordings. Our model offers means for noise reduction, targeted extraction of specific activation components in experimental designs, and potential integration across different recordings.

## 1 Introduction

Reliable individual-level measures of neural activity are essential to characterize interindividual variability in brain activity patterns, link such patterns with observed behaviors, and use them to support clinical decision making. In response to tasks or stimuli, magnetoencephalography (MEG) provides millisecond-precise measures of brain activation that display high consistency within an individual (e.g.,Ala-Salomäki et al., 2021;Schaefer et al., 2004;Virtanen et al., 1998). These capabilities make MEG a valuable tool for understanding individual differences in brain function. However, to fully leverage its potential to quantify individual variability in brain activity, new analysis approaches are needed, capable of systematically extracting reproducible, subject-specific brain activation patterns while retaining the accuracy of the measure.

Currently, in MEG studies, response consistency is generally assessed across a group of participants, which allows to gain statistical power at the cost of sacrificing individual specificity. These processes often largely discard the temporal and spatial resolution of the MEG signals where much of the individual variability would show. It is not easy to define a comparison method between different measures that retains the data’s temporal and spatial information without significant simplifications. Thus, features that may be highly consistent across measurements within an individual, but variable across the whole group, may remain unidentified.

Independent component analysis (ICA) (Jutten and Herault, 1991) has proven to be an efficient tool for analyzing electrophysiological data, and is currently widely used, e.g., in isolating physiologically distinct signals (e.g.,Delorme et al., 2012;Vigário et al., 2000), especially in the case of artifacts, from MEG recordings (Jung et al., 1998;Vigário et al., 1997). ICA decomposes the multivariate MEG signals into two distinct mathematical objects: one representing the temporal information and the other the spatial information. When these two objects are combined, they reconstruct the original data. Such a decomposition can greatly aid in comparing different signals by addressing the spatial and temporal information separately. Different ICA approaches have been proposed for analyzing multiple datasets simultaneously, as summarized, for instance, byHyvärinen (2013). These approaches tend to focus either on extracting a collective decomposition of the datasets based on assumptions on how similarly the spatial (Brookes et al., 2011;Hyvärinen, 2011) or the temporal information (Eichele et al., 2011;Richard et al., 2020) is shared among them, or on applying ICA independently to the different datasets and then comparing the decompositions. A limiting factor in all these methods is the lack of a comprehensive criterion for comparing the extracted decompositions. Approaches that address this problem typically focus either only on spatial or only on temporal information (e.g.,Himberg et al., 2004;Hyvärinen, 2011;Meinecke et al., 2002) and leave unanswered the question of how the other variable, whether temporal or spatial, behaves.

Here, we introduce a combined ICA method (comICA) for analyzing different MEG recordings simultaneously. ComICA combines temporal and spatial information comparisons to extract only those parts of the signal that are present in all recordings. We restrict our scope to different recordings of the activity evoked in response to the same stimuli and in the same subject. The underlying premise is that the time courses of evoked brain responses are highly reproducible within an individual. Here, the reproducibility of the time courses captured by MEG is interpreted as the brain responses being temporally identical up to independent additive noise. We propose that using this constraint and statistical analysis of the corresponding spatial information yields a principled method for extracting reproducible responses in different MEG recordings, accounting for similarities in both the spatial and temporal behaviors.

This paper describes the proposed algorithm and explores its effectiveness and limits first using simulated MEG data. We then demonstrate the ability of this method to extract reproducible responses in real MEG data recorded during a high-level cognitive task, picture naming, performed by the same participants on two different days. We show that this approach isolates common task-related activation at the sensor level in individual participants and eliminates noncondition-specific brain activation.

## 2 Methods: Mathematical Framework

We assume the following general underlying model for the MEG data: Task- or stimulus-related brain responses can be represented as a set of spatially stable features associated with feature-specific time courses (Delorme et al., 2012;Vigário et al., 2000). Moreover, the same spatial and temporal features can model the responses to the same task and stimuli in any other recording of the same participant. We extract this shared representation using ICA.

**Fig. 1.**
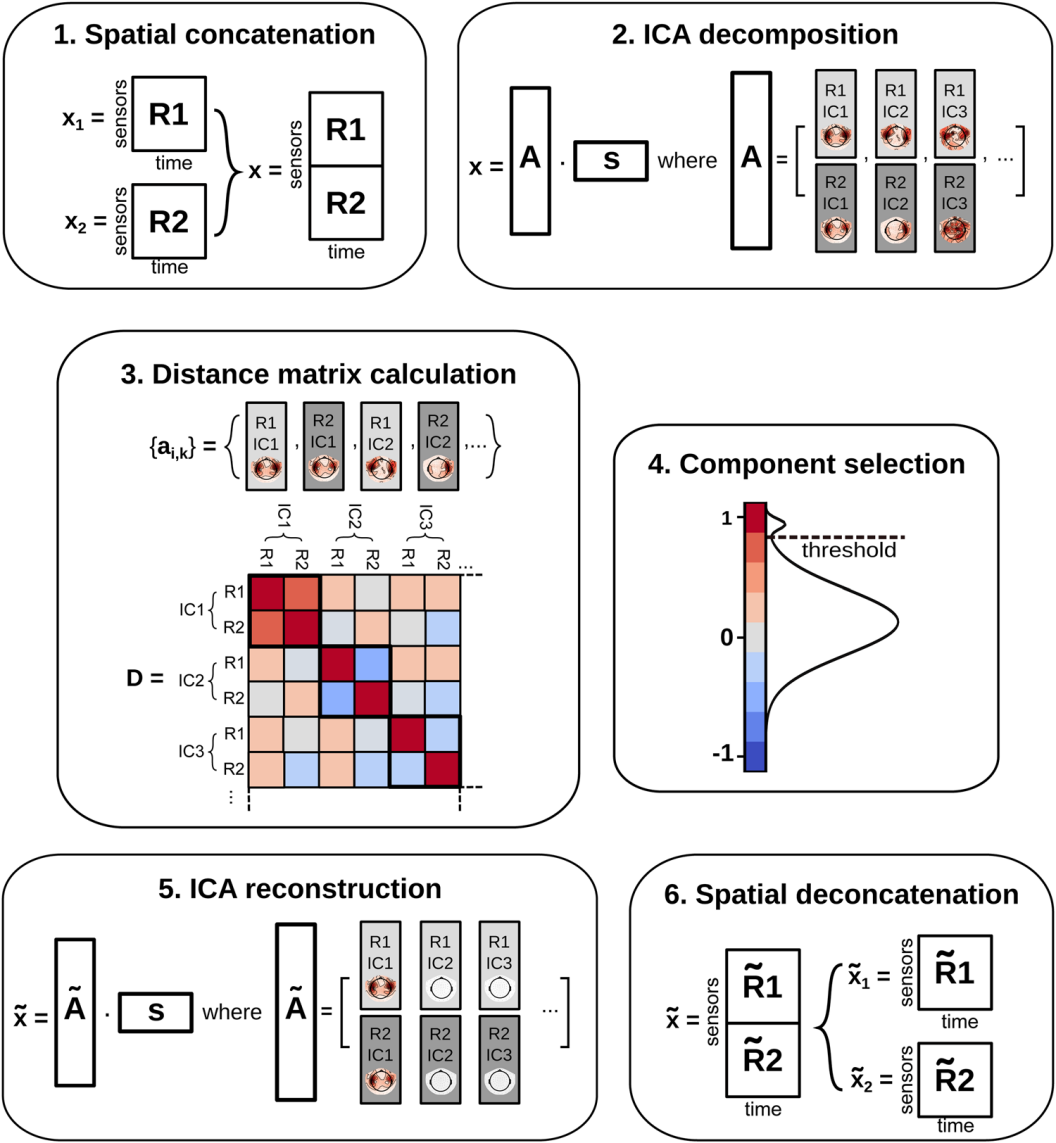
The comICA workflow illustrated in the case of two recordings (R1andR2). (1) Concatenation of the data along the spatial axis; (2) the resulting object is decomposed via ICA; (3) the recording-specific spatial coefficients {${\text{a}}_{i,k}$} are analyzed in the distance matrixD; (4) discarding those pairs of spatial coefficients that are not similar enough across the two recordings — i.e., those for which the block diagonal elements are not all above the threshold; (5) the data are reconstructed on the retained components; (6) the processed data for the two recordings are then obtained by splitting the resulting dataset into$\tilde{R}1$and$\tilde{R}2$.

We characterize the extracted components in terms of temporal and spatial information, applying constraints separately to both coordinates. A set of components is defined as shared when their temporal projections are identical and their spatial projections are statistically similar.Figure 1illustrates the comICA workflow in the case of two recordings.

The data decomposition is performed by first concatenating the recordings across the spatial dimension and applying ICA to the concatenated recordings. This first step involves extracting features from the data that are temporally shared between the different recordings. Let our data be$\text{x}={\left\{{\text{x}}_{1},{\text{x}}_{2},\ldots ,{\text{x}}_{M}\right\}}^{T}$, a set ofMrecordings. In the case of MEG, each${\text{x}}_{i}$is a matrix where each row corresponds to a measurement channel, i.e., spatial location, and each column to a time point$t_{k}$. Each${\text{x}}_{i}$should have the same dimensionality:$n_{sens}\cdot \bar{T}$, where$n_{sens}$is the number of sensors, and$\bar{T}$is the number of time points in the recording. The data structurexcomprises the single recordings along the spatial axis, resulting in a matrix of dimensions$(M\cdot n_{sens})\times \bar{T}$. Step 1 inFigure 1illustrates this concatenation in the case ofM=2recordings.

We then decomposexvia temporal ICA as

x=A⋅s, (1)

whereAis the mixing matrix of dimensions$(M\cdot n_{sens})\times$N, andsis the array ofNindependent components (ICs), or ICA sources, which has dimensionality$N\times \bar{T}$. Concatenating the data before the ICA decomposition results in a single set of sources shared across all recordings. This implies that the temporal information for all components is enforced to be identical in each recording, and the respective linear coefficients inAsolely account for any recording-specific spatial deviations.

Theith column of matrixArepresents the concatenatedMsets of spatial patterns relative to theith temporal source, as shown in Step 2 ofFigure 1. These spatial patterns can be interpreted as magnetic field patterns at the sensorlevel and do not depend on time.

To evaluate the similarity between spatial patterns across recordings, we extract all the recording-specific spatial patterns from the mixing matrixA, as illustrated in Step 3 ofFigure 1. Theith column ofAhas dimension$M\cdot n_{sens}$and can be separated intoMcontiguous subsets of length$n_{sens}$to obtain the recording-specific spatial patterns. For theith column ofA, this process defines the set${\left\{{\text{a}}_{i,k}\right\}}_{k=1}^{M}=\left\{{\text{a}}_{i,1},{\text{a}}_{i,2},\ldots {\text{a}}_{i,M}\right\}$. Here, each${\text{a}}_{i,k}$represents the spatial coefficients relative to thekth recording for theith independent component. By iterating over each column, we construct the set$\left\{{\text{a}}_{i,k}\right\}$, whereidesignates the column (or the independent component), ranging from 1 toN, andkdesignates the recording, spanning from 1 toM. For the sake of notation, we can reindex this set to be$\left\{{\text{a}}_{j}\right\}$wherejnow spans from 1 toM×N.

We quantify the similarity between any pairh,jof spatial patterns as

$$d_{h,j}=\frac{{\bar{\text{a}}}_{h}^{T}{\text{C}}^{+}{\bar{\text{a}}}_{j}}{\sqrt{{\bar{\text{a}}}_{h}^{T}{\text{C}}^{+}{\bar{\text{a}}}_{h}}\sqrt{{\bar{\text{a}}}_{j}^{T}{\text{C}}^{+}{\bar{\text{a}}}_{j}}},$$ (2)

which is the Euclidian cosine similarity between any pair of centered elements${\bar{\text{a}}}_{i}$and${\bar{\text{a}}}_{j}$, and the metric tensor${\text{C}}^{+}$is the pseudoinverse of the covariance matrixCof the space of the centered coefficients$\left\{{\bar{\text{a}}}_{j}\right\}$:

$$\text{C}=\frac{1}{2\cdot N}\sum_{i,k}\left({\text{a}}_{i,k}-\bar{\text{a}}\right)\left({\text{a}}_{i,k}-\bar{\text{a}}\right)=\frac{1}{2\cdot N}\sum_{i,k}{\bar{\text{a}}}_{i,k}\cdot {\bar{\text{a}}}_{i,k}.$$ (3)

It is important to note that the empirical estimation of the covariance matrix is sensitive to the number of samples, i.e., how many independent components and how many recordings we are analyzing.

The values of$d_{i,j}$obtained according toEquation 2are limited to the interval[−1,1], where$d_{i,j}=1$indicates${\bar{\text{a}}}_{i}$is parallel to${\bar{\text{a}}}_{j}$, i.e., the two spatial patterns are the same except for a positive scaling factor,$d_{i,j}=-1$indicates they are antiparallel, and$d_{i,j}=0$indicates that the two spatial patterns are uncorrelated.

We order the set$\left\{{\bar{\text{a}}}_{i,k}\right\}$so that the spatial patterns relative to the same componentifor the different recordings are contiguous, as portrayed in Step 3 ofFigure 1. The similarity measure expressed inEquation 2is computed for each possible pair of elements in$\left\{{\text{a}}_{i,k}\right\}$, and the results are arranged in a distance matrixD, where theNblock-diagonal elements$B_{i}$of dimensionM×Mcontain the distances of the spatial maps relative to the same independent component across the different recordings. Theith component is considered reproducible across theMrecordings if all the entries in the corresponding block$B_{i}$are above a certain threshold. This positive threshold can be chosen according to the desired significance level, as illustrated inFigure 1, Step 4. Ultimately, theith component is deemed reliable if all the corresponding block$B_{i}$entries are significantly close to 1.

A projected version of the concatenated data can be reconstructed from the components deemed reliable; Step 5 ofFigure 1for the case ofM=2recordings. Specifically, the ICA decomposition is inverted to reconstruct a new concatenated object$\tilde{\text{x}}$from the reliable components. The reconstructed object is then split intoMseparate datasets${\left\{{\tilde{\text{x}}}_{i}\right\}}_{i=1}^{M}$representing the reliable features in the original recordings; Step 6 ofFigure 1forM=2.

The comICA pipeline has been implemented in Python, and it provides a workflow compatible with the MNE-Python software package (Gramfort et al., 2013). The implementation of comICA studied in this paper utilizes the FastICA version of ICA (Hyvärinen, 1999), which is readily available in the MNE-Python software package.

## 3 Methods: Data

### 3.1 Simulated MEG data

A dataset was generated to explore the algorithm’s ability to separate neural sources with distinct activation time courses. To this end, MEG data were simulated for the 306-channel Vectorview system (MEGIN Oy, Espoo, Finland), which comprises 102 sensor triplets (two planar gradiometers and one magnetometer) arranged in a helmet-shaped array. The forward model was constructed based on the anatomical information of one subject from a previously published study (Ala-Salomäki et al., 2021). The cortical surface was reconstructed from the subject’s T1-weighted structural MRI using the FreeSurfer software package (Dale et al., 1999;Fischl et al., 1999) and decimated for a cortical source space with an average spacing of 9.9 mm between the source points. Neural activation at each source point was modeled by a current dipole normal to the local cortical surface. A single-compartment boundary-element model (BEM) based on the inner skull surface was employed to estimate the volume-current contribution to MEG (Gramfort et al., 2013).

In each iteration of the simulations, three randomly selected sources in the left hemisphere were activated, producing evoked responses. Two MEG recordings, or runs, were generated per iteration. Each run contained one source shared across the runs and one unique, as pictured in the lower part ofFigure 2. Altogether$N_{trials}=60$trials were simulated for each run, and multivariate Gaussian noise was added to each trial independently. The source activations were generated as Morlet wavelets and parametrized with respect to three features: amplitude (α), latency ($t_{0}$), and duration (T), as illustrated in the upper half ofFigure 2. The amplitude was defined as the peak-to-baseline distance of the signal, the latency as the time between the stimulus trigger and the peak of the source timecourse, and the duration was defined as the time span from the signal onset until the end of the simulated evoked response.

**Fig. 2.**
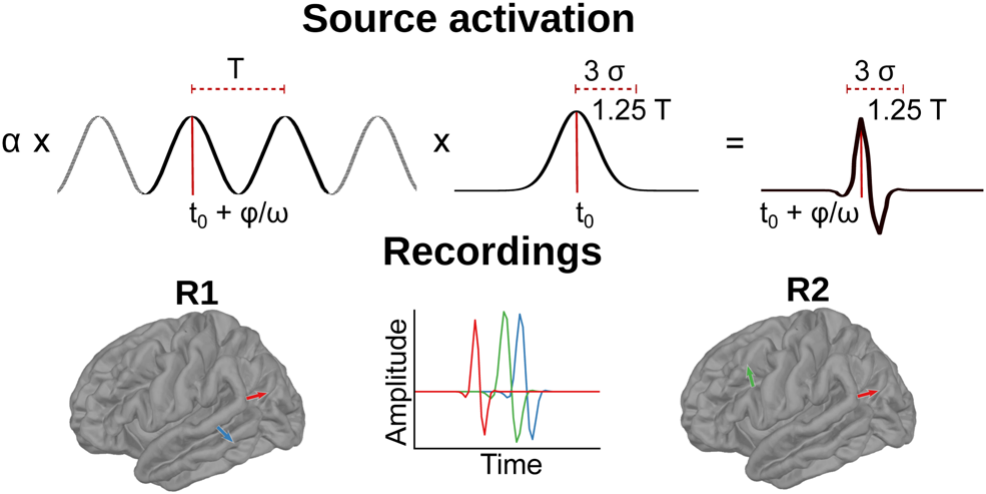
Sources constructed for the simulated data. Two neural sources were simulated for the two runs (R1 and R2) of each iteration. The evoked activity was modeled for each neural source as a Morlet wavelet. Each run contained one unique source (blue in R1 and green in R2) and one source that was shared between the two runs (red in both R1 and R2), for a total of three source activations per iteration.

The shape of the simulated signal was defined as

$$s\left(t\right)=\alpha \cdot \text{cos}\left(\omega \left(t-t_{0}\right)+\phi \right)\cdot \text{exp}\left(-\frac{{\left(t-t_{0}\right)}^{2}}{2\cdot \sigma^{2}}\right),$$ (4)

whereσ=0.375⋅Tand$\omega =\frac{3}{4}\cdot 2\pi /T$, andϕis a phase shift used to construct the shape of the source activation function. A constant value ofϕ=1​/​3⋅πwas used throughout the simulations, and all other parameters were uniformly randomized within the ranges indicated inTable 1.

**Table 1.** Ranges of the simulation parameters.

Variable	Min.	Max.
α	40 nAm	70 nAm
T	95 ms	500 ms
$t_{0}$	$\frac{T}{2}+50\text{ms}$	1150ms−T​/​2

The analysis was restricted to the gradiometer signals. The signals were sampled at$f_{s}=200\;\;\text{Hz}$. Time windows of−0.2, 1.2s were extracted around the activation onsets and averaged across trials. The time window was chosen to contain all the possible activation functions in the simulations fully. The simulations and preprocessing of the data were performed with MNE-Python version 0.23.4 (Gramfort et al., 2013).

Reproducible activation patterns were then extracted from the two runs. The data simulated in each run were concatenated along the spatial axis, resulting in one set of$2\cdot n_{sens}$time series of length$\left(1.2\cdot f_{s}\cdot N_{trials}\right)$time points. The concatenated dataset was centered to have zero mean before applying the ICA decomposition.

Temporal ICA was performed with the FastICA implementation on the concatenated dataset, and three components were extracted for each iteration. The covariance matrix weighting was not applied inEquation 2because this low number of independent components, only 3, was insufficient to calculate a reliable estimate for the covariance matrix. The components were instead selected using an unweighted cosine distance and a fixed threshold of 0.9. To assess the accuracy of the reconstruction, we computed the goodness of fit (GoF), as implemented in MNE-Python, by comparing the extracted shared source with the simulated ground truth at the time of the peak activation of the simulated source. The simulation comprised 10,000 iterations, resulting in a total of 20,000 GoF values.

### 3.2 Real MEG data

#### 3.2.1 Data description

The ability of the algorithm to extract common activations was further tested on MEG data recorded on two separate days from participants performing a picture-naming and visual processing task (Ala-Salomäki et al., 2021).

The data were measured at the MEG Core of Aalto NeuroImaging Infrastructure (Aalto University, Espoo, Finland) using a Vectorview whole-head MEG device (MEGIN Oy, Espoo, Finland) comprising 306 sensors (204 gradiometers and 102 magnetometers) arranged in a helmet-shaped array. Only gradiometer data were used in the analysis. Individual MRIs were acquired at the Aalto NeuroImaging Advanced Magnetic Imaging (AMI) Centre with a Magnetom Skyra 3.0T MRI scanner (Siemens GmbH, Erlangen, Germany) using a standard T1-weighted gradient-echo sequence. The dataset included 20 healthy human participants (10 females, 10 males; mean age 25 years; SD 3.9; age range 21–35 years) for whom the MEG evoked responses and oscillatory activity have been analyzed previously (Ala-Salomäki et al., 2021). The original authors collected the data after obtaining a written informed consent from all participants, in agreement with the prior approval of the Aalto University Research Ethics Committee. As in the original paper, one participant’s data were not used for our analysis due to noncompliance with the task instructions.

In the present study, we focused on a subset of two tasks from the original study: a picture-naming task, where the participants were presented with pictures and asked to name the depicted object overtly after a short delay, and a visual task, where the participants were shown pictures and were asked to respond occasionally to a target stimulus. MEG data were recorded from each participant, presenting 100 stimuli per condition, in two separate measurement sessions (1–13 days apart, mean 4.2, SD 3.9 days, total of 200 stimuli per condition per subject). The tasks and measurements details are described in full in the original paper (Ala-Salomäki et al., 2021).

#### 3.2.2 Preprocessing

Initial interference suppression was performed using the signal space separation method (Taulu and Simola, 2006) with a 16-s temporal window, a subspace correlation limit of 0.98, an inside expansion order of 8, and an outside expansion order of 3. According to the preprocessing steps by the original authors, the individual MEG data were transformed to a common head position within each recording and each subject (MEGIN Maxfilter software package; version 2.2.12).

The data were band-pass filtered to0.1−45Hz and resampled at 200 Hz. Artifacts related to eye movements and heartbeat were repaired by applying ICA to the MEG data and manually selecting the components that most resembled the artifact waveforms. Epochs from −0.2 s to 1 s around the stimulus onsets were extracted. All the data processing was performed using the MNE-Python package version 0.23.4 (Gramfort et al., 2013).

#### 3.2.3 Analysis of evoked responses from the two recordings

After rejecting data segments with the peak-to-peak signal amplitude exceeding$3\cdot 10^{-16}\text{T}/\text{m}$, indicating the presence of artifactual signals, the number of epochs per condition in each subject was equalized over the two recordings. The number of epochs for the two conditions was similarly distributed across the subjects and recordings (median for picture naming: 93; median for visual task: 95).

The epochs were averaged for each subject, recording, and condition separately. The evoked responses from the two recordings were then concatenated along the spatial dimension to obtain the concatenated datasets${\text{x}}_{\text{sbj},\text{cond}}$for each condition and subject. The ICA decomposition was performed with the FastICA algorithm on${\text{x}}_{\text{sbj},\text{cond}}$without performing any dimensionality reduction.

Independent components with a shared behavior between the two recordings that exceeded the chosen significance level (α=0.05) were selected for further analysis. A new concatenated dataset was constructed using the retained components and separated into two recordings. Hereafter, the data from the reconstructed datasets are referred to as the comICA-reconstructed evoked responses.

#### 3.2.4 Reconstructed evoked responses

The cortical sources corresponding to the reconstructed and original evoked responses were computed using the dSPM variant (dynamic statistical parametric mapping;Dale et al., 2000) of cortically-constrained L2 minimum-norm estimation (MNE), as in the original publication (Ala-Salomäki et al., 2021). The comICA-reconstructed evoked responses were corrected with respect to the baseline before source reconstruction.

The forward model was constructed in the same manner as in the simulations. In addition, a loose orientation constraint of 0.3 and a depth weighting exponent of 0.8 were applied. The noise covariance matrix utilized in the source estimation was calculated from the unprocessed, unaveraged 200-ms prestimulus baseline intervals of both tasks, separately for each measurement session. The dSPM MNEs of the individual subjects were then morphed onto a standard template brain (“fsaverage” of FreeSurfer;Dale et al., 1999;Fischl et al., 1999) with spatial smoothing and subsequently averaged at the group level.

Following the analysis pipeline by Ala-Salomäki and colleagues, the source-level amplitudes of the group averages were averaged across four successive time windows (0−200,200−400,400−600, and600−800ms) with respect to the stimulus onset. As in the original study, a custom-made cortical parcellation based on the Destrieux template (Destrieux et al., 2010;Fischl et al., 2004) was used for spatial averaging. Analogously to the original work (Ala-Salomäki et al., 2021), the group-averaged visual and picture-naming tasks were contrasted, and p-values were extracted against the null hypothesis of the two tasks inducing the same activations to determine significant brain activation in the different cortical parcels and time windows.

## 4 Results

### 4.1 Simulations

We were able to efficiently isolate shared sources in the simulated data, as presented inFigure 3(a). The median goodness-of-fit (GoF) across all simulated iterations was 93.95%. The overall distribution of the GoF in the simulated data was skewed toward high success values but presented a fat tail toward lower values of the success measure. This behavior indicates that even though the median GoF is good, failures in the reconstruction are possible.

**Fig. 3.**
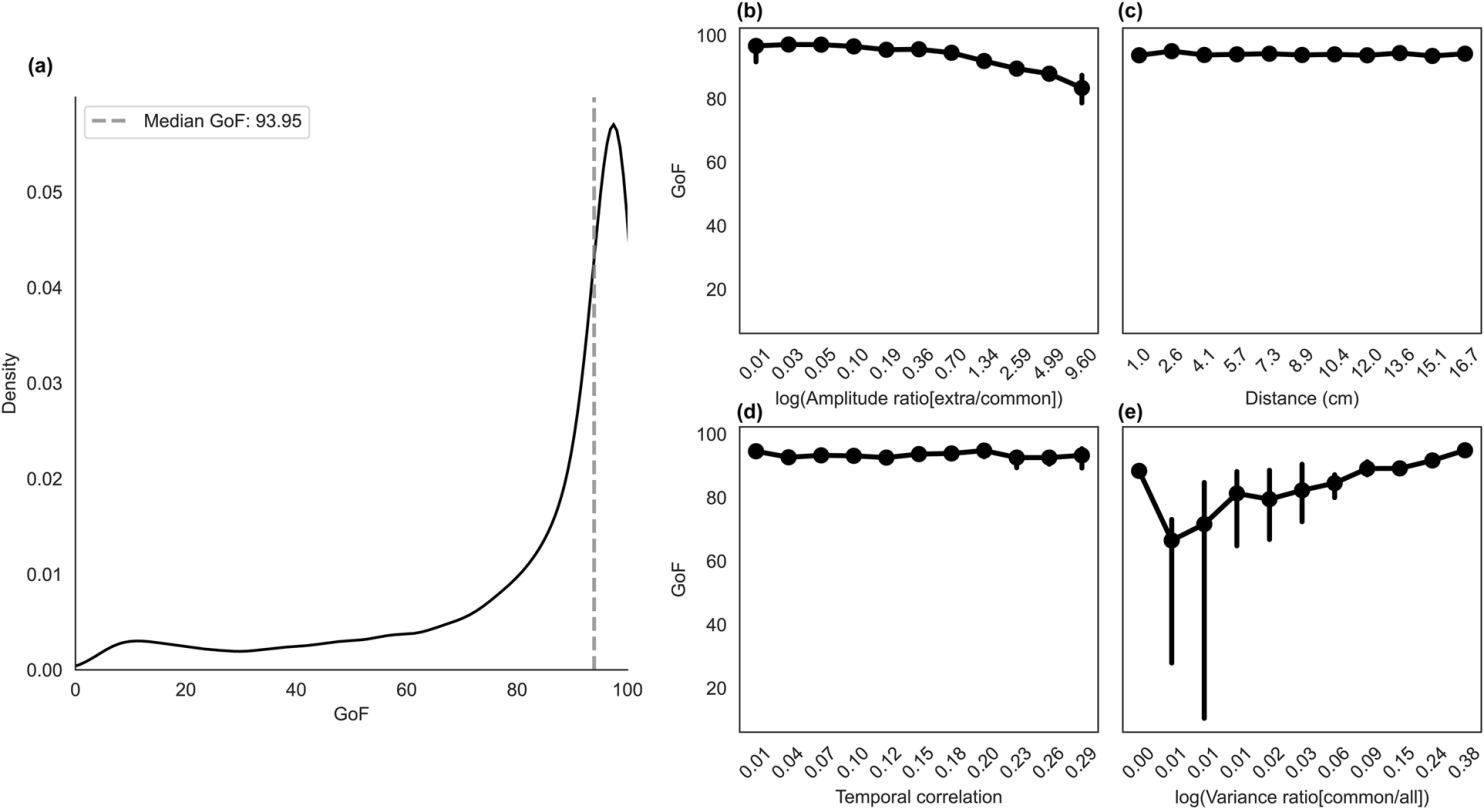
ComICA results for the simulated MEG data. (a) Probability distribution of the success in separating the shared sources in the simulated data. The distribution of the goodness of fit (GoF, expressed in percent) reveals the effective isolation of common sources. (b) to (e): Factors that may influence comICA identification of the shared source. (b) Amplitude ratio at the sensor level between the extra source and the common one, (c) distance between the sources, (d) temporal correlation of the sources, and (e) the ratio between the variance at the sensor-level data due to the common source only vs. both sources. In panels (b) to (e), the metric (y-axis) is the median GoF for the interval, reported with 95% confidence limits.

Figure 3(b)-(e)shows the effect of different run-specific variables on the ability to extract the shared source.Figure 3(b)presents the effect of the amplitude ratio of the shared vs. nonshared source at the sensor level, demonstrating that the shared source can be reconstructed more accurately when the amplitude of the signals generated by the shared source is higher than that of the unique source. Conversely, when the unique source produces stronger signals, the ability to reconstruct the shared source decreases monotonically.

The distance between the simulated sources — calculated as the shortest-path distance along the surface — did not significantly impact the ability to separate the brain sources, as depicted inFigure 3(c).

Figure 3(d)illustrates the effect of the temporal correlation between the time courses of the shared and nonshared sources when reconstructing the shared source. The temporal correlation between the time courses did not clearly affect the ability to isolate the single-source signal, as quantified by the median GoF. However, a higher temporal correlation between the source time courses resulted in a small increase in the variance of the ability to reconstruct the correct brain source accurately.

When the signals generated by the shared source explained a higher percentage of the overall variance of the run, the reconstruction of the shared source reconstruction improved (seeFig. 3(e)). Conversely, when the signals generated by the shared source explained a smaller percentage of the overall variance of the data, the ability to isolate and reconstruct the shared source decreased. Lower percentages of variance explained in the sensor-level signal by the source we aim to separate further led to an increase of the variance in the separation efficiency.

Overall, these findings indicate that source separation using comICA is achievable with good success for signals that simulate brain activation. However, the algorithm’s performance may be sensitive to variations in the contribution of shared vs. nonshared sources to the overall variance. It is important to note that the results presented here pertain to an ideal condition where the same signal was present identically in both recordings, exactly at the same location.

### 4.2 Real MEG data

After assessing the applicability of comICA on simulated data, we tested the method on a MEG test–retest dataset that had been previously analyzed for estimating reproducibility using intraclass correlation analysis (ICC) at the group level (Ala-Salomäki et al., 2021). As detailed in the Methods section, the comICA algorithm was applied independently on each subject.

**Fig. 4.**
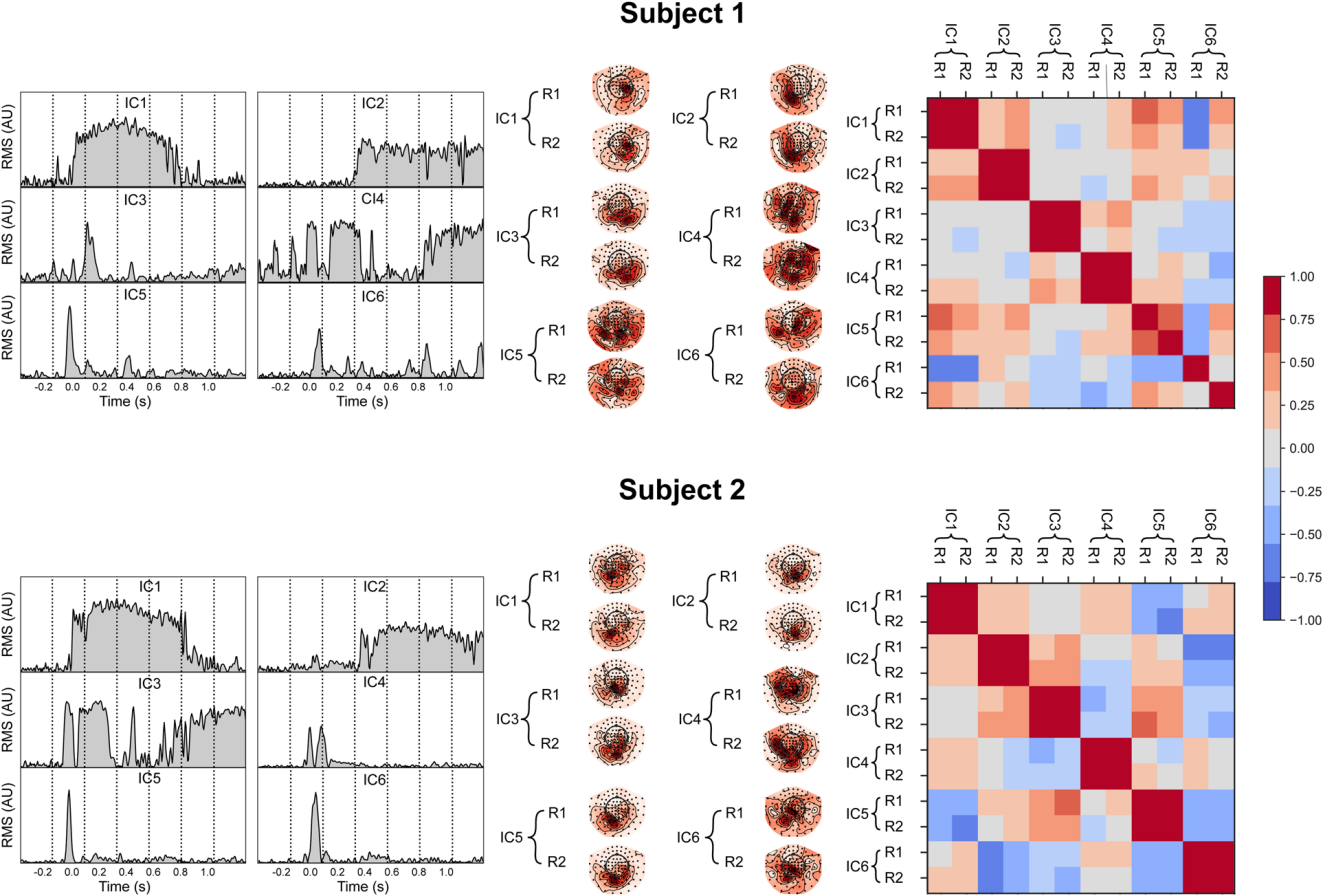
Visualization of comICA processing of the real MEG data. Left panel: The first six independent temporal components, ordered by the variance they explain, are presented for two subjects. Middle panel: Field patterns of the temporal components on the two recordings (R1 and R2) grouped by component (C). Right panel: Cosine similarity matrices for the spatial patterns. The block-diagonal elements in the matrices represent the similarity between the spatial representations of each component on the two different recordings, with higher similarity values (red) indicating more similar topographic activation maps.

Figure 4illustrates the intermediate outcomes for two individual subjects (Subject 1 and Subject 2). The independent components were extracted from the concatenated data of the two recordings for each subject and then ordered based on the percentage of variance they explained in the concatenated dataset. The first six components are shown here as an example. Notably, although the extraction process is done independently for each individual, in the leftmost column ofFigure 4, one can observe the salient pairwise correspondence between the IC time courses of the two subjects (e.g., C1–C1, C2–C2, C4–C3).

**Fig. 5.**
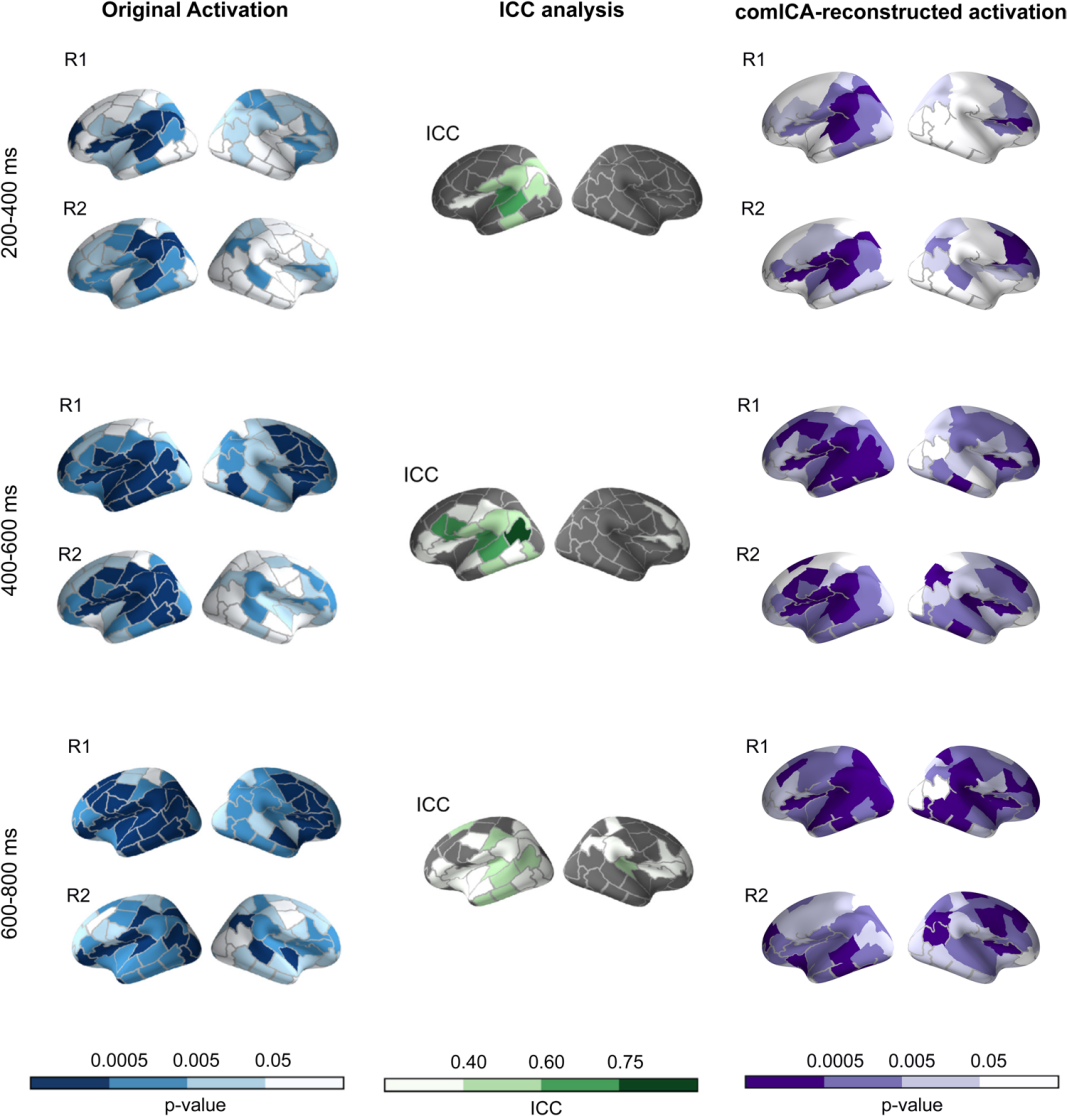
Group-level results on the real MEG data with and without comICA processing. Left: Group-level maps of activation for the contrast picture naming vs. visual task on day 1 and day 2 (R1,R2) on the original data (without comICA), as analyzed earlier byAla-Salomäki et al. (2021). The color scale represents the p-values against the null hypothesis (no difference between picture naming and visual task) in different parcels, averaged over the specified time windows. Middle: ICC values for the original data (no comICA applied). Darker color indicates higher consistency. Gray parcels were excluded from the ICC analysis as they did not meet the significance criteria (p-value < 0.005 for both recordings). Right: Group-level activation maps for the contrast picture naming vs. visual task on day 1 and day 2 on the comICA-reconstructed data. The color scale represents the p-values against the null hypothesis (no activation) in different parcels, averaged over the same time windows as the original data.

In each subject, the field patterns are highly similar across the two recordings for most of the components (similarity above 0.75, as quantified in the distance matrices). The red block-diagonal structure, depicted in the right-most column ofFigure 4, further illustrates the similarity of the spatial maps across the two recordings of each subject.

However,Figure 4also demonstrates some notable individual variability. For Subject 1, the first four components met the selection criteria for replicability, as indicated by the solid red diagonal blocks in the distance matrix. The fifth component could be selected at lower thresholds. In contrast, the sixth component had close-to-zero similarity between the spatial representations, as indicated by the gray off-diagonal elements. For Subject 2, all six components had a high degree of similarity. These degrees of similarity agree with the visual inspection of the corresponding spatial maps in the middle column.

Even though the time courses of the independent components display similarity between the two subjects, the field patterns corresponding to these similar time courses show apparent differences between the two subjects. This behavior points to possible interindividual variation in cortical processing, especially in the spatial aspect of the signals.

The reproducible part of the MEG signals for the contrast picture naming vs. visual task had been previously estimated using group-level ICC (Ala-Salomäki et al., 2021). To verify that the present comICA-derived results generally agree with the earlier ICC-based results, we first extracted shared field patterns from the test–retest data for each individual and each experimental condition (seeFig. 4). Source activity present in only one recording — likely irrelevant to the shared task between recordings — was discarded. Subsequently, the group-level source-space patterns of reproducible brain activity were calculated by averaging over the comICA-processed single-subject data and contrasting the picture-naming vs. visual-task conditions.

The group-level ICC analysis performed by the original authors highlighted significant similarities across the recordings in the left hemisphere. The comICA-reconstructed activation aligned with this result, as shown inFigure 5, outlining consistent left-lateralized activation patterns, especially in the early time window (200–400 ms). With the comICA approach, also notable right-hemisphere activation remained in the later time windows.

## 5 Discussion

In the present study, we introduced the comICA algorithm for extracting shared activations across two (or more) MEG recordings of evoked responses. The algorithm is based on ICA paired with selection rules considering temporal and spatial information. Specifically, comICA extracts temporally identical independent sources and applies a principled spatial criterion for their selection. To evaluate the performance of this method, we conducted tests using simulated data, which provided metrics to quantify its precision. Additionally, the application of comICA on real MEG data yielded results compatible with previous reports, further showcasing the potential applications of our method to single-subject brain recordings.

### 5.1 Simulations

When evaluating the ability of comICA to separate the signals originating from simulated sources that were shared between two MEG recordings vs. unique to a single recording, high levels of separability were achieved, as indicated by the algorithm’s high median goodness of fit of 93.95%. Upon further investigation, some of the characteristic parameters of the simulated signals were found to influence the success rate of such separability.

A typical concern in ICA is that temporally correlated time courses violate the model assumption (Hyvärinen and Oja, 2000), and we, therefore, expected correlated sources to be hard to separate. Indeed, we found that sources with maximally uncorrelated time courses resulted in better separation overall. In cases where two sources were more correlated, a wider variability in the success rate of comICA was observed. However, a high median success rate was still reached even with this broader spread of the success rate. This suggests that breaking the assumption of temporal independence behind ICA — by focusing on temporally correlated time courses — does not imply a clear decline in the performance of comICA in separating two time courses.

The amplitude ratio at the sensor level, i.e., how much of the variance of the combined dataset could be attributed to each source, was a predictor of high GoF. Sources contributing little to the dataset’s overall variance were not generally successfully separated. These sources generally generate lower signal amplitude at the sensor level when compared with the other source in the data. This result suggests that brain responses with significant amplitude differences at the sensor level, or those generally contributing little to the data variance, can be harder to separate than two comparable signals with temporally correlated time courses. This is possibly due to the PCA preprocessing behind the applied ICA algorithms, but no tests have been made with implementations of ICA that did not rely on this step. Future work should investigate this aspect further. However, one possible implication of this result is that comICA is better able to isolate consistent high-SNR signals than sources characterized by weaker signals compared with the noise. This, in particular, would apply to the case where comICA is used to separate signals from high-amplitude sources of noise. For example, this seems to be a pressing issue in removing artifacts such as those induced by TMS, where ICA applications are common (e.g.,Atti et al., 2024;Rogasch et al., 2017;Wu et al., 2018).

Although MEG has limitations in separating nearby sources (Hari et al., 1988), especially when their activation time-courses overlap, the spatial proximity of the simulated sources had minimal impact on source separability. This finding underlines the primary importance of the temporal information of the signals in algorithms that heavily rely on temporal ICA.

Thus, even though ICA has many limitations (Hyvärinen and Oja, 2000), both dependent and independent of the algorithm utilized (as summarized, for instance, bySariya and Anand (2018)), the performances of comICA in separating the shared sources in two recordings of evoked responses showed consistent success on the simulated data. It is worth noting that the use of ICA is typically discouraged on evoked responses due to their nonstationary nature (e.g.,Metsomaa et al., 2017). In our work, we did not directly address this issue. Instead, we tested the reliability of the results regardless of it and sought to quantify the primary controllable sources of error. The overall success of the algorithm in this scenario seems to hint that the general nonstationarity issue of ICA does not significantly affect the performance of comICA.

### 5.2 Real MEG data

In the picture-naming dataset, the brain activity that comICA isolated is interpreted as the one that is shared across the two recordings performed on different days. The comparison of the group-level results obtained on data with vs. without the application of comICA shows that the comICA processing and potential dimensionality reduction do not affect the overall behavior of the signals of interest. Notably, the experimental contrast of interest was preserved for the comICA-reconstructed vs. original data.

Importantly, owing to the ability of ICA to factorize temporal and spatial information by observing the different components selected in each subject by comICA, individual differences can be exposed and understood separately in terms of space or time. For instance, inFigure 4, temporal components from different subjects showed similar time courses. However, the field patterns corresponding to these comparable temporal components exhibited notable differences across the subjects. This behavior suggests that shared temporal behaviors in evoked responses could be extracted, at least to an extent, via comICA even across subjects, similarly to previous approaches (Eichele et al., 2011;Richard et al., 2020). Still the time courses across subjects were similar, but not identical, especially for the ones with sustained temporal activation. This would imply that methods that work at the group level may discard a good part of the components if trying to extract them as shared for a group. Moreover, these extracted components at the group level would be excluded during the component selection process outlined here due to their spatial differences. Intuitively, our results support the idea that different subjects, when presented with the same stimulus, are more likely to share a high degree of similarity in the shape of their temporal responses than in their field patterns. Anatomical differences only may not be enough to account for such high variability, and future work may be needed to investigate this individual variation in more depth.

### 5.3 Methodological considerations

By applying ICA to the concatenated datasets, the temporal information of the shared sources was constrained to be identical for both recordings. This approach ensures that the selected sources share the same temporal behavior. It is important to note that imposing this temporal constraint makes comICA only suitable for time-locked activation, hence repeating identically across the different recordings. Moreover, the salient individual differences revealed by comICA, especially in the spatial domain, emphasize that comICA primarily applies to one subject at a time.

The metric chosen to select the similar spatial patterns was designed to behave like a cosine similarity, enabling our algorithm to overlook differences in the intensity of the same temporal source in the two recordings. The field patterns intuitively indicate the location of the temporal signal in the sensor space. Therefore, choosing a cosine-similarity measure aims to retain signals with the same spatial activation patterns while not discriminating on intensity differences. Because these intensity differences in the spatial coefficients can be different for different independent components, after applying comICA, the reconstructed signals need not be identical for the two (or more) recordings.

Importantly, the selection criteria applied to the field patterns rely on selecting components based on the overall distribution of the spatial distance measure. The threshold in the selection process was determined by analyzing the pairwise distances across all extracted components. Setting a limit based on this distribution is only reasonable when the independent component analysis generates enough components to estimate their overall distribution. Investigating other thresholding methods compatible with this case was out of the scope of the present analysis.

The simulation results demonstrated the promising performance of comICA, showcasing its ability to meet our desired levels of accuracy for brain-like signals. However, it is essential to note that the simulated data only tested specific waveforms and, therefore, are not necessarily representative of more general cases. However, we consider our selection of waveforms to be meaningful within the context of MEG evoked responses. Additionally, a good alignment of the findings obtained from real MEG data with the previous literature further supports our interpretation of the results.

The main limiting factor in separating shared components from different recordings appears to be the similarity in the variances of the different components in the signal. This allows comICA to still perform optimally, for instance, when isolating meaningful brain activity from a recording that contains stimulus-locked artifacts, provided the intensity of the artifact is comparable with that of the signals of interest. In such cases, comparing the contaminated recording with a similar reference which does not contain the same artifact could aid in extracting shared brain signals while excluding artifacts, or, more generally, noninteresting components.

Although the analysis presented in this paper relies solely on the FastICA implementation of ICA, the performances of the other algorithms implemented in MNE-python, i.e., Infomax (Bell and Sejnowski, 1995) and Picard (Ablin et al., 2018), were also compared on the simulated data, but no significant difference was observed in the results.

### 5.4 Conclusions

In this work, we showed that we can identify shared brain responses in MEG datasets at the sensor level when we define such common activation as a combination of the same time courses and statistically indistinguishable spatial coefficients across the sensors. We claim this result can be used to isolate task-specific brain activation in two recordings from the same subject. In real recordings of complex brain activity, such as that of picture naming, comICA performed well in extracting the activations shared between the two recording sessions. Given comICA’s assumptions, we expect it to perform equally well for EEG data or any other electrophysiological recordings characterized by signals of high temporal accuracy.

## Data Availability

In agreement with the ethical permission and national privacy regulations at the time of the study, the raw MEG data cannot be made openly available. The derived results that served as input for the figures will be made openly available. The libraries developed for the analysis described in this paper are openly available athttps://github.com/AaltoImagingLanguage/comICA.git.
